# Prognostic Significance of Vascular Endothelial Growth Factor Serum Determination in Women with Ovarian Cancer

**DOI:** 10.5402/2012/245756

**Published:** 2012-06-26

**Authors:** Elisabetta Bandiera, Roberta Franceschini, Claudia Specchia, Eliana Bignotti, Chiara Trevisiol, Massimo Gion, Sergio Pecorelli, Alessandro Davide Santin, Antonella Ravaggi

**Affiliations:** ^1^Division of Gynecologic Oncology, “Angelo Nocivelli” Institute of Molecular Medicine, University of Brescia, 25123 Brescia, Italy; ^2^Department of Clinical Pathology, Regional Center for Biomarkers, Azienda ULSS 12, 30122 Venice, Italy; ^3^Department of Biomedical Sciences and Biotechnology, Medical Statistics Unit, University of Brescia, 25123 Brescia, Italy; ^4^Istituto Oncologico Veneto IOV-IRCCS, 35128 Padova, Italy; ^5^Department of Obstetrics and Gynecology and Reproductive Sciences, Yale University School of Medicine, New Haven, CT 06511, USA

## Abstract

*Introduction.* We performed a review of the literature to elucidate the potential prognostic significance of serum vascular endothelial growth factor (sVEGF) levels in ovarian cancer. *Methods.* Eligible studies in English and Italian were identified in MEDLINE/PubMed from VEGF discovery to October 2011. All studies evaluating: (i) sVEGF levels before any surgical and chemotherapeutic treatment; (ii) the association between sVEGF levels and the established prognostic variables; (iii) the value of sVEGF levels in predicting patients' outcomes, were selected for this review. *Results.* The search resulted in 758 titles. Nine studies met the inclusion criteria. A statistically significant association between the level of sVEGF and FIGO stage, tumour grade, residual tumour size, lymph node involvement, and presence of ascites was found in at least one study. sVEGF, in comparison with the established prognostic factors, appears to be the best prognostic marker for overall survival, since it stands out as an independent prognostic factor in most of the studies considered. Moreover, sVEGF levels were shown to be independent prognostic factors by 2 out of the 3 studies that considered DFS as an end point. *Conclusion.* High levels of sVEGF identify a subgroup of patients with higher risk of death and/or recurrence. These patients should be eligible for individually tailored therapeutic interventions.

## 1. Introduction

Ovarian cancer is the most frequent cause of death from gynaecological cancer and the fourth most frequent cause of cancer-related death in women in Europe and the United States [1]. It has the highest fatality-to-case ratio of all gynaecological malignancies, mainly due to the fact that it is characterized by early widespread metastasis and high-grade malignancy at diagnosis. The five-year survival proportion is about 80–90% for patients with stage I disease and only 15–20% for patients with stage III or IV disease. Although survival has improved with the use of maximal cytoreduction surgery along with platinum- and taxane-based chemotherapy, nearly 80% of ovarian cancers relapse and patients inevitably succumb to the development of chemotherapy-resistant disease [2].

Clinicopathological features known to be prognostic variables for ovarian cancer are surgical stage (FIGO stage), histological grade, lymph node involvement, residual tumour size after cytoreductive surgery, histological subtype, ascites, and age. According to the three-year analysis of the FIGO Annual Report on the Results of Treatment in Gynaecological Cancer, stage, grade, and residual tumour size have the greatest prognostic value [3]. However, these factors provide an insufficient picture of the biology of ovarian cancer and they are frequently interrelated. The identification of new serological biological markers that predict the outcome of the disease would be extremely useful for developing individually tailored and possibly more effective treatments. Serum analysis is a noninvasive technique feasible in cases where no tissue is available and it can also be performed during followup.

It is well established that angiogenesis, the formation of new blood vessels, is necessary for the growth and metastatic spread of solid tumours [4–7]. A high degree of tumour angiogenesis has been shown to correlate with poor survival in women with ovarian cancer [7–10]. Vascular endothelial growth factor (VEGF) plays an essential role in angiogenesis in many tumour types [11–15]. It is a heparin-binding dimeric glycoprotein involved in angiogenic, mitotic, and microvascular permeability-inducing activities, leading to extravasation of plasma proteins and proangiogenic stromal changes [16].

Several studies have found VEGF levels to be significantly higher in the tissues and biological fluids of women with ovarian cancer compared with healthy controls [17–20], whereas its association with tumour progression and/or patient survival is still controversial. We performed a review of the literature to elucidate the prognostic role of serum VEGF (sVEGF) levels in ovarian cancer, both alone and in comparison with established clinicopathological factors.

## 2. Methods

Eligible studies in English and Italian were identified in MEDLINE (PubMed version) from VEGF discovery to October 2011 using the terms VEGF, “vascular endothelial growth factor” and synonyms, “ovarian cancer,” “ovary cancer,” and synonyms. We searched studies that used these terms in title and abstract and that was indexed by bibliographic database with the Mesh term “ovarian neoplasms.” We searched the database using these terms separately and also in combination.

Relevant papers were independently selected by two of the reviewers (E. Bandiera and R. Franceschini) based on the following inclusion criteria: studies that evaluated (i) sVEGF levels before any surgical and chemotherapeutic treatment; (ii) the association of sVEGF levels with the established clinicopathological prognostic factors (FIGO stage, tumour grade, residual tumour size, lymph node involvement, histological type, ascites, age); (iii) the value of sVEGF levels in predicting patients' outcomes (overall survival (OS), disease-free survival (DFS), progression free survival (PFS)). Any disagreement in the inclusion of one study between two reviewers was solved by discussion.

### 2.1. Data Extraction

Two of the reviewers (E. Bandiera and R. Franceschini) independently reviewed each study and abstracted data on first author, country of study, study characteristics (study design, followup duration, therapy), clinical and pathological variables, and study outcomes.

## 3. Results

### 3.1. Included Studies

Our search strategy identified 758 journal abstracts. From these, we retrieved for further evaluation 15 full-text articles focused on the relationship between circulating preoperative VEGF and prognosis in ovarian cancer. Of these 15 articles, nine [21–29] studies met the inclusion criteria and were used for this review. Hefler et al. [28] gathered together some cases from trials by Tempfer et al. [21], Gadducci et al. [22], Chen et al. [23], and Cooper et al. [25]. Because Hefler et al. [28] added a series of new patients, we reported these five [21–23, 25, 28] studies independently.

### 3.2. Excluded Studies

Six studies were excluded. The paper by Manenti et al. [30] was excluded because these authors analysed plasma VEGF levels. Since VEGF is secreted also by platelets, Manenti et al.'s study was unsuitable for comparison with studies focusing on sVEGF. Boss et al. [31], Bamias et al. [32], Yamamoto et al. [33], and Rudlowski et al. [34] were excluded because they did not directly evaluate the prognosis of patients with abnormal sVEGF levels and/or the association between sVEGF levels and clinicopathological characteristics. Finally, Dirix et al. [35] evaluated patients with different cancer types and did not report independent results for ovarian cancer.

### 3.3. Characteristics of the Selected Studies

The nine selected studies were published between 1996 and 2010 and included 529 patients from seven countries. All but one [28] study were retrospective. Six of these nine studies reported details on the duration of followup (median: 42 months [21], 34 months [23], 29 months [29] or mean: 39 months [28], or length: 60 months [25], 24 months [22]). All studies analysed the association between sVEGF levels and the established prognostic variables and evaluated the association between sVEGF levels and OS. Three studies [21, 23, 27] analysed the association between sVEGF and DFS whereas only one [29] analysed the association between sVEGF and PFS.

### 3.4. Characteristics of Study Populations

All studies enrolled women with newly diagnosed and histopathologically confirmed ovarian cancer except the one by Cooper et al. [25], which included also a small group of women with peritoneal and fallopian tube malignancies (Table 1). The mean (or median) ages of the studied women ranged from 52.5 to 64 years. Most cancers (>95%) were epithelial and the predominant histological type was serous carcinoma. Most patients were diagnosed with poorly differentiated ovarian cancer, advanced FIGO stage, and ascites. With the exception of Gadducci et al. [22], who followed only 27 patients with advanced disease receiving chemotherapy, all other studies monitored all patients enrolled.

Surgery for optimal tumour debulking included hysterectomy, bilateral salpingo-oophorectomy, omentectomy [21, 23, 28, 29], pelvic and para-aortic lymphadenectomy [21, 23, 28, 29], and appendectomy [23, 28, 29]. Five studies [22, 24–27] did not describe the surgical approach, but according to the reported data (residual tumour size [22, 24–27], omental metastasis [26], lymph node involvement [26]), we may presume that maximal cytoreductive surgery was performed. 

Surgery was followed by chemotherapy consisting of platinum analogues alone [21–24, 28] or in combination with taxane [25, 29]. Early stages of disease were treated according to the standards established by the respective institutions: patients with stage IA-IB [21], I-II [22], IA [25], and IA-IB excluding clear cell histology [28] did not receive any chemotherapy or were treated like patients with advanced disease [23, 24, 29]. Although postoperative chemotherapy is the accepted standard treatment, two studies [26, 27] omitted any information about it.

### 3.5. sVEGF Assay

The sVEGF assay method was similar across studies. Venous blood was taken preoperatively from all patients. All sera were separated and stored at ≤20°C. Seven studies [21–25, 27, 28] used the same Quantikine sandwich ELISA kit (R&D Systems Minneapolis, USA). Li et al.'s study used a home-made indirect ELISA kit, whereas Mahner et al.'s study used VEGF-165 ELISA KIT (Siemens Healthcare Diagnostic, Tarrytown, USA).

Data on the precision of sVEGF assays were reported in three studies [21, 24, 28], and in all studies, the intra/inter-assay coefficient of variation was <10%. Median values of sVEGF reported by authors were: 466 pg/mL [21], 229 pg/mL [22], 458 pg/mL [23], 440 pg/mL [24], 379 pg/mL [25], 387 pg/mL [27], 407 pg/mL [28], and 171 pg/mL [29]. Li et al. [26] showed a mean value of 765 pg/mL.

### 3.6. Relationship between sVEGF Levels and the Other Prognostic Factors

The association between sVEGF concentrations and FIGO stage, tumour grade, residual tumour size, lymph node involvement, histological type, ascites, and age was analysed by 89%, 89%, 89%, 44%, 67%, 56%, and 78% of studies respectively (Table 2).

When the median (or mean) of sVEGF values was evaluated in relation to clinicopathological features, a statistically significant association between the level of sVEGF and FIGO stage, tumour grade, residual tumour size, lymph node involvement, and presence of ascites was found in at least one study. By contrast, no statistically significant association was found between sVEGF levels and histological type or age.

Tempfer et al. [21], Chen et al. [23], and Li et al. [26] demonstrated that elevated sVEGF levels were associated with a high malignant potential of tumours (G1 versus G2-G3 [21, 23], G1-G2 versus G3 [26]). Gadducci et al. [22], Cooper et al. [25], and Li et al. [26] reported a positive association between sVEGF concentrations and ascites volume (Gadducci et al. [22] selected patients with stages III-IV). Li et al. [26] and Hefler et al. [28] found that patients with suboptimally debulked cancer had higher sVEGF values than patient in whom tumour debulking was optimal. Finally, only the study by Li et al. [26] showed that sVEGF values were higher in patients with advanced FIGO stages and lymph node involvement.

### 3.7. sVEGF Evaluation

In evaluating its association with outcome variables, the levels of sVEGF were dichotomised using different cut-offs: 75th percentile [21, 23] or median [24, 29] in ovarian cancer patients, mean [26] in healthy subjects, and 95th percentile [27] in patients with benign disease. In one study, the authors used the value maximizing the hazard ratio [25]. Cut-offs ranged from 100 to 826 pg/mL. Finally, only Hefler and coworkers [28] considered sVEGF as a continuous variable.

### 3.8. Statistical Analyses

In the univariate analyses, seven [21, 23, 24, 26–29] studies used the Kaplan-Meier product-limit method to estimate how sVEGF and other clinicopathological variables might predict OS and DFS. Gadducci et al. [22] and Cooper et al. [25] did not explicitly report the method of univariate analysis. In the multivariate analyses, all studies claim to have used the Cox proportional hazards regression model to assess the independent role of different, simultaneously evaluated prognostic factors in determining outcomes. Estimates are reported in terms of relative risk (RR) and hazard ratio (HR). The results of univariate and multivariate analyses were considered statistically significant when the *P*  values were <0.05.

### 3.9. Univariate and Multivariate OS Analysis

Univariate and multivariate analyses for survival were reported in Table 3. All studies analysed the association between sVEGF levels and OS. With the exception of Gadducci et al. [22] and Mahner et al. [29], all authors found that elevated sVEGF was associated with shorter OS. Moreover, five [21, 23, 25, 27, 28] of these seven studies found sVEGF to be an independent prognostic factor.

As expected, clinicopathological features known to be prognostic variables for EOC such as FIGO stage, tumour grade, residual tumour size after cytoreductive surgery, lymph node involvement, and age have been shown as independent prognostic factors in at least one study. Notably, sVEGF, in comparison with others prognostic variables, was reported as independent prognostic factors by the majority of studies.

Chen et al. [23], Li et al. [26], and Hefler et al. [28] chose subgroups of patients for further analyses. Chen et al. [23] selected a subset of 40 patients with residual tumour size less than 2 cm. Univariate analysis, performed only for sVEGF, showed that elevated sVEGF was associated with shorter OS. Multivariate analysis identified sVEGF, FIGO stage, and grade as independent prognostic factors.

Li et al. [26] demonstrated that there was no significant difference in cumulative survival probability between stage I/II patients with high values of sVEGF and stage I/II patients with low levels of sVEGF. By contrast, the cumulative survival probability of stage III/IV patients with high values of sVEGF was lower than that of stage III/IV patients with low levels of sVEGF.

A planned subgroup analysis was performed for 56 patients with FIGO stage I in the study by Hefler et al. [28]. In univariate analysis, only sVEGF was associated with OS. In multivariate analysis, sVEGF and tumour grade were independent prognostic factors for OS.

### 3.10. Univariate and Multivariate DFS and PFS Analysis

Only three [21, 23, 27] of the nine included studies considered DFS as an end point. In univariate analysis, a significant association between DFS and sVEGF level was found by 2 [21, 23] out of 3 [21, 23, 27] studies. In multivariate analysis, sVEGF levels were shown to be independent prognostic factors by 2 [21, 23] out of 3 [21, 23, 27] studies. The associations between DFS and other prognostic factors were shown in Table 4.

Chen et al. [23] further evaluated DFS for 40 ovarian carcinoma patients with residual tumour size less than 2 cm, and they found that elevated sVEGF levels were significantly associated with lower DFS in univariate analysis and sVEGF levels, FIGO stage and grade were independent prognostic factors for DFS in multivariate analysis.

Finally, only Mahner et al. [29] considered PFS as an end point and he did not find a significant association between PFS and sVEGF level.

## 4. Discussion

The management of patients with ovarian cancer is based on established prognostic factors such as tumour stage, histological grade, and residual tumour size after cytoreductive surgery. Recently, the concept of standard chemotherapeutic treatment with platinum/taxane combination, the necessity of adjuvant chemotherapy in early stages of disease, the use of neoadjuvant chemotherapy for patients expected not to be optimally debulked at primary cytoreductive surgery and the use of consolidation chemotherapy for patients at high risk of recurrence have all been questioned.

The need for additional prognostic data to calibrate therapeutic tools on an individual basis in women with ovarian cancer seems obvious. In contrast to other malignancies, no serological prognostic parameter other than CA125 has been shown to have clinical value in ovarian cancer, even though CA125 serum levels at diagnosis are not associated with OS and DFS [36, 37].

From VEGF discovery till 2011, nine studies that directly correlated preoperative sVEGF with ovarian cancer outcome were published. Structured data extraction was performed on the articles to compare study populations, sVEGF assays, associations between sVEGF and clinicopathological characteristics, patient management, and outcome evaluation. Unfortunately, because of the heterogeneity of the studies and missing or incomplete information, it is not possible to pool data and to perform a meta-analysis in order to obtain univocal indications about sVEGF's prognostic value.

The data reported in Tables 1, 3, and 4 show evident differences among studies. All studies included only epithelial ovarian carcinoma patients except those by Cooper et al. [25] and Li et al. [26], where other ovarian, peritoneal, and tubal malignancies were included. Patients underwent different chemotherapy regimens based on platinum analogues alone [21–24, 28] or in combination with taxane [25, 29]. Patients with early-stage disease were treated differently or were not treated, depending on the standards of the respective institutions. None of the studies exhaustively described followup (time, lost patients, events). Although seven [21–25, 27, 28] out of nine studies used the same sVEGF assay, one [22] of these measured significantly lower sVEGF values in ovarian cancer patients. Finally, widely differing sVEGF cut-off values (ranging from 100 to 826 pg/mL) were chosen for univariate and multivariate analysis, depending on the statistical methods chosen for the analysis.

In order to find out how sVEGF influences ovarian cancer biology, all studies analysed the association between sVEGF and the clinicopathological characteristics of the patients. The results seem to confirm that VEGF plays an important biological role in the pathogenesis of ascites [38, 39]. VEGF increases vessel permeability for circulating macromolecules, thus facilitating extravasation of a plasma-rich exudate into the peritoneal cavity. Moreover, seven out of eight studies, concerning the relationship between sVEGF and FIGO stage, showed that VEGF concentrations measured in sera were not associated with FIGO stage. This may indicate that the effects promoted by VEGF are a continuous process and are independent of the clinical progression of the disease.

Interestingly, in our review of literature, sVEGF appears to be the best prognostic marker for OS in comparison with the established prognostic variables, since it stands out as an independent prognostic factor in most of the studies considered.

The scarcity of the data on the relationship between sVEGF levels and DFS makes it difficult to draw any firm conclusions in this regard. However, it is worth noting that sVEGF appears to be an independent prognostic factor for DFS in 2 out of 3 studies, as well as tumor stage and grade.

Chen et al. [23] and Hefler et al. [28] analyzed the prognostic value of sVEGF in a selected “low-risk” group of patients. Chen et al. [23] showed that sVEGF, FIGO stage and tumour grade were independent prognostic factors for OS and DFS in 40 patients with size tumour less than 2 cm. Hefler et al. [28] in a cohort of 56 patients with FIGO stage I found that sVEGF and tumour grade were independent prognostic factor for OS. The value of these results is conspicuous in those situations where the usefulness of adjuvant chemotherapy or the advisability of more chemotherapy cycles for certain categories of patients is under discussion.

In conclusion, almost all of the studies analysed in the present review, including the largest one by Hefler et al. [28], showed that elevated levels of sVEGF were significantly associated with shorter OS. It is worth noting that multiple phase III studies, ICON 7, GOG218, and OCEANS, have recently showed that the use of bevacizumab, a humanized antibody against VEGF, provides a clinically meaningful benefit in EOC patients outcome [40, 41].

Thus, from analysis of the literature reported in this review, as well as from results reported by recent clinical trials, sVEGF appears to be a promising prognostic factor in ovarian cancer that could identify a subgroup of patients with poor survival and higher risk of death that could benefit of bevacizumab therapy to improve their outcome.

## Figures and Tables

**Table 1:** Clinical and demographic characteristics of patients.

Author, year										
		Tempfer et al., 1998 [21]	Gadducci et al., 1999 [22]	Chen et al., 1999 [23]	Oehler and Caffier, 2000 [24]	Cooper et al., 2002 [25]	Li et al., 2004 [26]	Harloziňska et al., 2004 [27]	Hefler et al., 2006 [28]	Mahner et al., 2010 [29]
Origin and Dates		1990–1995 Austria	1990–1997 Italy	1992–1998 Taiwan	Germany	1995–2000 Iowa	1999–2001 China	1997–2002 Poland	1990–2003 Austria, Iowa, Italy, Taiwan	1996–2004 Germany

Number of cases		60	53	56	41	101^$^	50	86	314	37

Age, mean or median* (range), years		55.6* (36–71)	59* (23–81)	52.5* (21–88)	62 (32–83)	64 (20–78)	NA	NA	59.9	58.61* (26–78)

FlGO stage	I	7	19	14	5	20	17	14	56	1
	II	12	1	6	2			9	27	1
	III	27	22	32	30	81	33	37	177	29
	IV	14	11	4	4			26	46	6

Grade	G1	21	17	21	NA	39	13	19	60	0
	G2	39	12	35	NA			41	88	9
	G3		24		NA	61	30	26	150	27

Epithelial ovarian cancer	Serous	28	33	30	32	81	15	49	166	31
	Mucinous	23	6	4	2		9	4	41	0
	Undifferentiated	3	6	8	0		19	13	15	4
	Endometrioid	3	7	10	7		0	20	39	1
	Clear cell	2	0	4	0	0	0	0	9	1
	Others	1	1	0	0	0	0	0	44	0

Nonepithelial ovarian cancer		0	0	0	0	0	7	0	0	0

Ascites	>500 mL/presence**	NA	21**	NA	NA	67**	22	64**	NA	21
	<500 mL/absence**	NA	12**	NA	NA	34**	28	22**	NA	15

Residual disease	<2 or 1*** cm	38	9	40	11	68***	44	32	164***	22
	>2 or 1*** cm	22	24	16	30	33***	6	54	64***	14

Lymph node involvement	yes	23	NA	NA	NA	NA	12	NA	38	10
	no	37	NA	NA	NA	NA	38	NA	63	11

^$^: Cooper study contains a small group of peritoneal and fallopian tube malignant cancers; NA: not available data; *: Median values; **: Numbers of patients with presence/absence of ascites; ***: Numbers of patients with residual disease < or >1 cm.

**Table 2:** Association between sVEGF and clinicopathological characteristics of patients.

Variable	Author, year	No. cases	Reported statistics for VEGF	Variable type	Statistical significance of association
Stage	Tempfer et al., 1998 [21]	60	md	I/II versus III/IV	NO
	Gadducci et al., 1999 [22]	53	md	I versus II and III versus IV	NO
	Chen et al., 1999 [23]	56	md	I/II versus III/IV	NO
	Oehler and Caffier, 2000 [24]	41	m	categorical	NO
	Cooper et al., 2002 [25]	101	md	I/II versus III/IV	NO
	Li et al., 2004 [26]	50	m	I/II versus III/IV	YES
	Harloziňska et al., 2004 [27]	86	NA	I/II versus III/IV	NO
	Hefler et al., 2006 [28]	314	m	categorical	NO

Grade	Tempfer et al., 1998 [21]	60	md	G1 versus G2/G3	YES
	Gadducci et al., 1999 [22]	53	md	G1-G2 versus G3	NO
	Chen et al., 1999 [23]	56	md	G1 versus G2/G3	YES
	Cooper et al., 2002 [25]	101	md	G1-G2 versus G3	NO
	Li et al., 2004 [26]	50	m	G1-G2 versus G3	YES
	Harloziňska et al., 2004 [27]	86	NA	G1 versus G2/G3	NO
	Hefler et al., 2006 [28]	314	m	categorical	NO
	Mahner et al., 2010 [29]	37	md	G2 versus G3	NO

Residual tumour size (cm)	Tempfer et al., 1998 [21]	60	md	≥2 versus <2	NO
	Gadducci et al., 1999 [22]	53	md	≥2 versus <2	NO
	Chen et al., 1999 [23]	56	md	≥2 versus <2	NO
	Oehler and Caffier, 2000 [24]	41	m	≥2 versus <2	NO
	Cooper et al., 2002 [25]	101	md	≥1 versus <1	NO
	Li et al., 2004 [26]	50	m	≥2 versus <2	YES
	Hefler et al., 2006 [28]	314	m	≥1 versus <1	YES
	Mahner et al., 2010 [29]	37	md	≥0 versus <0	NO

Lymph node involvement	Tempfer et al., 1998 [21]	60	md	yes versus no	NO
	Li et al., 2004 [26]	50	m	yes versus no	YES
	Hefler et al., 2006 [28]	314	m	yes versus no	NO
	Mahner et al., 2010 [29]	37	md	yes versus no	NO

Histological type	Tempfer et al., 1998 [21]	60	md	serous or mucinous versus others	NO
	Gadducci et al., 1999 [22]	53	md	serous versus others	NO
	Chen et al., 1999 [23]	56	md	serous or mucinous versus others	NO
	Oehler and Caffier, 2000 [24]	41	m	categorical	NO
	Li et al., 2004 [26]	50	m	categorical	NO
	Harloziňska et al., 2004 [27]	86	NA	categorical	NO

Ascites (mL)	Gadducci et al., 1999 [22]	53	md	presence versus absence	YES
	Cooper et al., 2002 [25]	101	md	presence versus absence	YES
	Li et al., 2004 [26]	50	m	≥500 versus <500	YES
	Harloziňska et al., 2004 [27]	86	NA	presence versus absence	NO
	Mahner et al., 2010 [29]	37	md	≥500 versus <500	NO

Age (years)	Tempfer et al., 1998 [21]	60	md	≥50 versus <50	NO
	Gadducci et al., 1999 [22]	53	NA	NA	NO
	Chen et al., 1999 [23]	56	md	≥50 versus <50	NO
	Oehler and Caffier, 2000 [24]	41	m	≥60 versus <60	NO
	Cooper et al., 2002 [25]	101	md	≥64 versus <64	NO
	Hefler et al., 2006 [28]	314	m	continuous variable	NO
	Mahner et al., 2010 [29]	37	md	≥61 versus <61	NO

Md: median, m: media, NA: not available data.

**Table 3:** Univariate and multivariate analyses for overall survival.

Variable	Author, year	No. cases	Cut-off	Univariate analysis RR or HR, *P*-value	Multivariate analysis RR or HR, *P*-value
VEGF (pg/mL)	Tempfer et al., 1998 [21]	60	≥826 versus <826	RR = 2.7, *P* = 0.007	RR = 2.7, *P* = 0.008
	Gadducci et al., 1999 [22]	53	NA	*P* = NS	NA
	Chen et al., 1999 [23]	56	NA	*P* < 0.001/*P* = 0.006*	RR = 4.47, *P* ≤ 0.001; RR = 5.37*, *P* < 0.001*
	Oehler and Caffier, 2000 [24]	41	≥440 versus <440	HR = 3.56, *P* = 0.026	*P* = NS
	Cooper et al., 2002 [25]	101	≥380 versus <380	HR = 2.13, *P* = 0.009	HR = 2.08, *P* = 0.02
	Li et al., 2004 [26]	50	≥100 versus <100	*P* = 0.0085; *P* = 0.4^§^; *P* = 0.02^∧^	*P* = 0.0750
	Harloziňska et al., 2004 [27]	86	≥750 versus <750	*P* = 0.0169	RR = 2.35; *P* = 0.02
	Hefler et al., 2006 [28]	314	continuous variable	*P* < 0.001; *P* < 0.001°	HR = 1.8, *P* = 0.03; HR = 1.1°, *P* = 0.001°
	Mahner et al., 2010 [29]	37	171	*P* = 0.302	NA

Stage	Tempfer et al., 1998 [21]	60	I/II versus III/IV	RR = 3.2, *P* = 0.007	RR = 3.2, *P* = 0.001
	Chen et al., 1999 [23]	56	I/II versus III/IV	NA	RR = 2.08, *P* = 0.11; RR = 3.84*, *P* = 0.01*
	Oehler and Caffier, 2000 [24]	41	I/II versus III/IV	HR = 2.24, *P* = 0.043	*P* = NS
	Cooper et al., 2002 [25]	101	I/II versus III/IV	HR = 10.15, *P* < 0.001	HR = 9.24, *P* < 0.001
	Li et al., 2004 [26]	50	NA	NA	*P* = NS
	Harloziňska et al., 2004 [27]	86	I/II versus III/IV	*P* = 0.0006	RR = 4.08, *P* = 0.008
	Hefler et al., 2006 [28]	314	NA	*P* < 0.001	HR = 1.7, *P* < 0.001

Grade	Tempfer et al., 1998 [21]	60	G1 versus G2/3	RR = 1.4, *P* = 0.005	RR = 1.4, *P* = 0.01
	Chen et al., 1999 [23]	56	G1 versus G2/3	NA	RR = 2.38, *P* = 0.034; RR = 2.44*, *P* = 0.045*
	Cooper et al., 2002 [25]	101	G1/2 versus G3	HR = 1.36, *P* = 0.29	HR = 0.86; *P* = 0.63
	Li et al., 2004 [26]	50	NA	NA	*P* = NS
	Harloziňska et al., 2004 [27]	86	G1 versus G2/3	*P* = 0.00079	*P* = NS
	Hefler et al., 2006 [28]	314	NA	*P* < 0.001; *P* = 0.2°	HR = 1.2, *P* = 0.3; HR = 3.4°, *P* = 0.02°

Residual tumor size (cm)	Chen et al., 1999 [23]	56	≥2 versus <2	NA	RR = 1.34, *P* = 0.46
	Oehler and Caffier, 2000 [24]	41	0 versus 1 + 2	HR = 11.68, *P* = 0.018	HR = 11.68, *P* = 0.018
	Cooper et al., 2002 [25]	101	0 versus 1 + 2	HR = 2.2, *P* = 0.007	HR = 1.29, *P* = 0.42
	Li et al., 2004 [26]	50	NA	NA	*P* = 0.019
	Harloziňska et al., 2004 [27]	86	≥2 versus <2	*P* = 0.00637	*P* = NS
	Hefler et al., 2006 [28]	314	≥1 versus <1	*P* < 0.001	HR = 1.8, *P* = 0.006

Lymph node involvement	Tempfer et al., 1998 [21]	60	Yes versus No	RR = 2.8, *P* = 0.0007	RR = 2.8, *P* = 0.006
	Li et al., 2004 [26]	50	NA	NA	*P* = NS

Histological type	Chen et al., 1999 [23]	56	serous/mucinous versus others	NA	RR = 0.99, *P* = 0.92; RR = 1.14*, *P* = 0.21*
	Li et al., 2004 [26]	50	NA	NA	*P* = NS
	Harloziňska et al., 2004 [27]	86	serous versus others	*P* = NS	*P* = NS
	Hefler et al., 2006 [28]	314	serous versus others	*P* = 0.3; *P* = 0.6°	HR = 1.1, *P* = 0.6; HR = 1°, *P* = 0.9°

Ascites	Cooper et al., 2002 [25]	101	presence versus absence	HR = 2.5, *P* = 0.004	HR = 1.28, *P* = 0.54

Age (years)	Oehler and Caffier 2000 [24]	41	≥60 versus <60	*P* = NS	*P* = NS
	Cooper et al., 2002 [25]	101	NA	HR = 1.34, *P* = 0.30	HR = 1.16, *P* = 0.63
	Harloziňska et al., 2004 [27]	86	≥62 versus <62	*P* = 0.0478	RR = 2.20, *P* = 0.0272
	Hefler et al., 2006 [28]	314	continuous variable	*P* = 0.01, *P* = 0.8°	HR = 1, *P* = 0.9; HR = 1°, *P* = 0.6°

*:Subset of 40 patients with residual tumour size ≤2 cm; °:Subset of 56 patients with stage I; ^§^:Subset of patients with stages I-II; ^*∧*^:Subset of patients with stages III-IV; NA: not available data; NS: non-significant statistical analysis.

**Table 4:** Univariate and multivariate analyses for disease free survival.

Variable	Author, year	No. cases	Cut-off	Univariate analisys RR, *P*-value	Multivariate analisys RR, *P*-value
	Tempfer et al., 1998 [21]	60	≥826 versus <826	RR = 1.8, *P* = 0.003	RR = 1.8, *P* = 0.02
VEGF (pg/mL)	Chen et al., 1999 [23]	56	NA	*P* = 0.001, *P* = 0.001*	RR = 3.34, *P* = 0.002; RR = 5.62*, *P* < 0.001*
	Harloziňska et al., 2004 [27]	314	≥750 versus <750	*P* = NS	*P* = NS

Stage	Tempfer et al., 1998 [21]	60	I/II versus III/IV	RR = 1.3, *P* = 0.01	RR = 1.3, *P* = 0.02
	Chen et al., 1999 [23]	56	I/II versus III/IV	NA	RR = 2.09, *P* = 0.10; RR = 3.28*, *P* = 0.027*
	Harloziňska et al., 2004 [27]	314	I/II versus III/IV	*P* = 0.000	RR = 4.66, *P* = 0.00018

Grade	Tempfer et al., 1998 [21]	60	G1 versus G2/G3	RR = 1.9, *P* = 0.03	RR = 1.9, *P* = 0.04
	Chen et al., 1999 [23]	56	G1 versus G2/G3	NA	RR = 2.24, *P* = 0.042; RR = 2.55*, *P* = 0.037*
	Harloziňska et al., 2004 [27]	314	G1 versus G2/G3	*P* = 0.0001	*P* = NS

Residual tumour size	Chen et al., 1999 [23]	56	≥2 versus <2	NA	RR = 0.96, *P* = 0.93
	Harloziňska et al., 2004 [27]	314	≥2 versus <2	*P* = 0.0001	*P* = NS

Lymph node involvement	Tempfer et al., 1998 [21]	60	Yes versus No	RR = 2.8, *P* = 0.009	RR = 2.8, *P* = 0.009

Histological type	Chen et al., 1999 [23]	56	serous/mucinous versus others	NA	RR = 0.97, *P* = 0.73; RR = 1.04*, *P* = 0.7*
	Harloziňska et al., 2004 [27]	314	serous versus others	*P* = NS	*P* = NS

Age (years)	Harloziňska et al., 2004 [27]	314	≥62 versus <62	*P* = NS	*P* = NS

*:Subset of 40 patients with residual tumour size ≤2 cm; NA: not available data; NS: non-significant statistical analysis.
